# Densification of coal fines and mildly torrefied biomass into composite fuel using different organic binders

**DOI:** 10.1016/j.heliyon.2019.e02160

**Published:** 2019-07-25

**Authors:** A.A. Adeleke, J.K. Odusote, O.A. Lasode, P.P. Ikubanni, M. Malathi, D. Paswan

**Affiliations:** a Department of Mechanical Engineering, University of Ilorin, Ilorin, Nigeria; b Department of Materials and Metallurgical Engineering, University of Ilorin, Ilorin, Nigeria; c Mechanical Engineering Department, Landmark University, Omu Aran, Nigeria; d Metal Extraction and Recycling Division, National Metallurgical Laboratory, Jamshedpur, India

**Keywords:** Metallurgical engineering, Coal dust, Briquettes, Blended binder, CCS, IRI, Densification

## Abstract

Coal processing industries generate millions of tons of fines (<3 mm) during mining operation and are often considered as wastes. These wastes have enormous potential in serving as energy and metallurgical operation feedstock. One avenue for its use is densification into briquettes or pelletizes. Various briquetting techniques have been adopted in the past few decades; however, the main issues upfront in commercializing these techniques are significant binder cost and poor mechanical integrity. Therefore, the present study concentrates on utilizing commonly available organic binder along with pretreated biomass in developing coal fine briquettes. Briquettes were produced after initial pretreatment of the raw materials under a load of 2 tons. Briquettes were cured in an inert environment and eventually characterized for its main litmus requirements (physical properties). It was observed that pitch-molasses bonded briquettes have better physical properties leading to good mechanical integrity than briquettes produced from individual binder. The proximate, ultimate and calorific value analyses of the briquettes do not deteriorate but mildly improved compared to the raw coal fines. With a density of 1.18–1.32 g/cm^3^, drop to fracture that is greater than 100 (times/2 m), impact resistance index well above 6000, water resistance index of 99% and cold crushing strength of 9 MPa, pitch-molasses bonded briquettes clearly surpassed recommended physical properties benchmarked for briquettes of industrial and domestic end use. The physical properties of the briquettes favorably meet requirements as feedstock for rotary kiln direct reduced iron and COREX iron-making processes as well as fuel for thermal operations.

## 1 Introduction

Coal is an important raw material utilized as sources of fuel and reductant in metallurgical industries [1]. High grade coking coals are preferred for metallurgical application especially in blast furnace operations, however, their availability are limited world widely. This has encouraged alternative routes that can accommodate lean grade coal as fuel and reductant for extraction of iron from its ore. Processes such as COREX iron making, direct reduced iron via Tunnel and Rotary kilns are examples of the alternative routes. The production, transportation and handling of lump lean grade coal inevitably lead to generation of large fines. It's been reported that millions tons of fines (<3 mm) are generated during mining operations [2, 3]. These fines are often regarded as wastes because they fail to participate in various processes when applied as fuel source or reductant. Utilization of these fines will not only add value to them but also serve as a means of reducing environmental pollution.

One way to utilize this waste is to briquette/pelletize it to size range adoptable as fuel and metallurgical reductant. Efforts have been made to produce briquettes from coal fines [1, 2, 4, 5, 6]. The results from these efforts have been reported to be suitable for different applications. However, the industrial application of such method to produce briquettes that meets metallurgical requirements has not been well established. This is evident as Tata steel rotary kiln section still largely requires solution to the menace of huge loss and high production cost due to large waste (<3 mm) generated from lean grade coal during production, transportation and handling [7]. This is due to the fact that binders utilized fail to retain its property in some temperature range leading to poor mechanical integrity of briquettes.

Biomass such as rice husk, palm fibers and shell had been incorporated into briquetting of bituminous coal fines to reduce NO_x_ and SO_x_ released during combustion of coal [2, 8]. Briquettes strength from the composite of coal and biomass has been significantly low and this may be due to inadequate understanding of an effective way to utilize biomass for an improved mechanical integrity. The use of raw biomass in conjunction with coal often leads to weaker solid fuel with drastically reduced calorific value [5]. This is due to the presence of high moisture content, voluminous volatile matters as well as lean energy content within the biomass. However, torrefaction process can be used to overcome this limitations and drawback. However, torrefaction process can be used to overcome this limitations and drawback. However, torrefaction process can be used to overcome this limitations and drawback. Torrefaction process, within its temperature range of operation (200-300°C),basically lead to hemicellulose and cellulose degradation as well as enacting the lignin content of biomass which makes it useful as fuel and also as binder [9, 10]. Therefore, the present study is focused on production of briquettes (composite fuel) with good mechanical integrity from lean grade coal fines and pretreated biomass.

## 2 Materials and methods

### 2.1 Materials

The raw materials utilized in this study are Jhama coal fines, biomass (melina wood), pitch, starch and molasses (23° 44ʹN, 86° 24ʹE). Coal fines less than 3 mm were dried under the sun, pulverized and screened to particle sizes below 0.70 mm. It was further oven-dried at 105°C for 30 min to remove moisture before blending. Melina wood was also pulverized into particle size less 2 mm using a Laboratory Mill (Thomas Wiley, Model 4) for initial pretreatment (torrefaction process).

### 2.2 Torrefaction of melina

Pulverized biomass (<2 mm) was subjected to torrefaction process at 260°C and resident time of 60 min [9, 10] in a tubular furnace. Inert environment was achieved by a continuous flow of Nitrogen at 2 L/min into the furnace chamber. The biochar obtained after torrefaction were pulverized and screened to particle size below 0.70 mm.

### 2.3 Production of briquettes

Coal fines (95 wt.%), biochar (5 wt.%) and binder were homogeneously mixed, after which water was added and stirred properly to activate the binders for agglomeration. The binders (pitch, molasses and starch) were varied in the range of 3–20% of the entire briquette weight. In order to improve the mechanical integrity of the briquettes after preliminary mechanical test using various binders, pitch (P) and molasses (M) were blended together in different ratios of B1 (5P-5M), B2 (8P-7M), B3 (10P-5M) and B4 (5P-10M). Typically, 8% pitch and 7% molasses in the blend is denoted by 8P-7M and similarly for other blends. The optimum amount of water adopted for the study was in the range 8–10%. Molasses and starch bonded briquettes required lesser amount of water than pitch for proper agglomeration as observed during the mixing process. Briquetting of 25 g of the blends was carried out in a 25 mm internal diameter steel die (at room temperature) under a hydraulic press machine with a closely fitting plunger at a pressure of 28 MPa. Briquettes were cured at room temperature for 24–36 h for initial moisture evacuation and then in an inert environment at a temperature of 300°C for 30–120 min.

### 2.4 Characterization of the raw materials and the briquettes

#### 2.4.1 Raw materials

The proximate analysis of biomass, biochar and coal fines were carried out according to IS:1350-1 [11] standards. The CHN analysis of the biomass and coal fines were carried out in a LECO-CHN628 Analyzer using ASTM D5373 [12] standard. The gross calorific values for biomass, biochar and coal fines were determined in a Parr 6200 Oxygen Bomb Calorimeter in accordance with ASTM D5865-04 s [13].

#### 2.4.2 Briquettes

(a) *Density:* The height, diameter and mass of green briquettes were measured and recorded in order to calculate the green density. The measurements were repeated after the briquettes were cured in order to obtain the dry density. The density was calculated as a ratio of mass to volume for each briquette. The volume of the samples was calculated using Eq. (1).

(1) $$\text{V}={\pi \text{r}}^{2}\text{h}$$

where V is the volume (${\text{cm}}^{3})$, r is the radius of the cylindrical samples (cm) and h is the height (cm). The density (ρ) was then obtained using Eq. (2);

(2) $$\rho =\frac{\text{M}}{\text{V}}$$

(b) *Cold crushing strength*: Cold crushing strength (compressive strength) was carried out on the samples using a universal mechanical testing machine (10 kN Hounsfield apparatus) located at National Metallurgical Laboratory, Jamshedpur, India. The machine was operated in a compression mode as applicable for coke and briquettes [14, 15, 16, 17]. Each briquette was placed between the crushing jaws and allowed to have a tight contact with pressure head at 0.5 mm/s strain rate. The maximum crushing load (${\text{M}}_{\text{f}}$) which the briquettes can withstand before cracking or breaking was recorded and repeated twice for each sample. The average was used to calculate CCS in accordance with Eq. (3). D is the bottom circular diameter of the briquettes.

(3) $$\text{CCS}=\frac{4{\text{M}}_{\text{f}}}{\pi {\text{D}}^{2}}$$

(c) *Drop to fracture and impact resistance*: During drop resistance test, 3 briquette samples were dropped from stagnant height of 2 m until it shatters. Then times per 2 m (times/2 m) were adopted to evaluate drop resistance. From the drop test, impact resistance index (IRI) was calculated using Eq. (4).

(4) $$\text{IRI}=\frac{100\times \text{Average}\;\text{number}\;\text{of}\;\text{drops}/2\text{m}}{\text{Average}\;\text{no}\;\text{of}\;\text{pieces}}$$

(d) *Water resistance*: A simple immersion-in-water test on a single briquette was adopted for resistance against absorption of water and disintegration (water resistance index-WRI) in accordance with modified Richard's method [18]. Briquette of a known weight (${\text{W}}_{1})$was immersed in a cylindrical glass containing 220 ml distilled water at 30 ± 2°C for 30 minutes after which it was removed and cleaned to remove water on its surface and reweighed (${\text{W}}_{2})$. Relative change in weight of the briquettes was measured and percentage water absorbed was calculated using Eq. (5) from which WRI (%) was evaluated with Eq. (6);

(5) $$\text{\%}\;\text{of}\;\text{water}\;\text{absorbed}=\;\frac{{\text{W}}_{2}-{\text{W}}_{1}}{{\text{W}}_{1}}\;\times 100$$

(6) WRI(%)=100−%watergained

(e) *Combustion properties and microstructural analyses*: The proximate ultimate and gross calorific value analyses were carried out on the briquettes based on ASTM standards. Microstructural and point elemental analyses were carried out in a scanning electron machine (FEGSEM) coupled with an energy dispersive x-ray (EDX) spectrometer.

## 3 Results and discussion

### 3.1 Ultimate, proximate and gross calorific value analyses of raw materials

The ultimate, proximate and gross calorific value analyses of parent biomass, biochar, coal and pitch are presented in Table 1. Table 1 shows that the pretreatment process (mild torrefaction) carried out on the biomass (melina) led to an improvement in its energy properties. Biochar is the product obtained from the torrefaction process and it has fixed carbon, carbon and gross calorific values higher than the raw biomass. The moisture content also reduced from 7.52 to 2.68%. The improvement in properties of biochar through torrefaction has also been reported in previous studies [19, 20, 21, 22]. The volatile matter of biomass, biochar and pitch is higher than that of coal. It implied that all of them will contribute to the volatile matter of the briquette to be produced. Biomass and biochar have lower ash contents compared to coal and this implied that it will contribute lesser ash to the composite fuel. The first major tests for fuel or metallurgical/energy briquettes are the ability to survive handling, transportation and storage treatments before they get to be used [1, 2, 14, 17, 23]. The result presented in this study highlighted the major physical properties that evaluate the handling, transportation and storage treatments. These properties include bulk density, cold crushing strength (CCS), impact resistance (IRI), drop to fracture and water resistance (WRI). The thermal properties such as proximate, ultimate and calorific values of some selected briquette samples are also reported and discussed.

**Table 1:** Ultimate, proximate and gross calorific analyses of raw materials.

Raw materials	Melina	Biochar	Coal	Pitch
Proximate analysis (wt. %, dry basis)				
MC	7.52	2.68	1.37	0.18
VM	81.42	54.07	13.77	73.99
AC	2.15	2.17	17.94	0.95
FC	8.92	41.08	66.92	24.88
Ultimate analysis (wt.% dry basis)				
C	47.09	66.05	73.43	89.17
H	6.65	5.18	2.51	7.43
N	0.38	0.30	1.31	0.30
S	0.19	0.20	0.71	0.50
O	43.54	26.30	22.04	2.60
GCV(MJ/kg)	18.39	23.45	23.13	39.73

### 3.2 Density

The bulk density of the green briquettes (initial) are in the range of 1.30–1.56 g/cm^3^ for all binders including the blend of pitch-molasses as presented in Table 2. The bulk density was reduced to a range of 1.18–1.32 g/cm^3^ for cured briquettes. The reduction was as a result of the curing process that leads to reduction in weight due to moisture loss and loss of some light volatiles in briquettes raw materials. There was no fine consistent pattern observed traceable to curing conditions for all binders. However, it can be observed that increasing holding time from 30 to 120 min causes a decline due to higher moisture loss and mild devolatilization which led to higher shrinkage. The range of 1.18–1.32 g/cm^3^ is higher than 0.87 g/cm^3^ of blast furnace coke reported by Mori et al. [24]. The ranges is a reminiscent of formed coke produced from Victorian brown coal by Mollah et al. [4]. It suggests tshat pore volume of cured briquettes is lower than that of green briquettes but lesser than that of blast furnace coke. It implied that there will be ease in transportation of the briquettes produced.

**Table 2:** Density of green and cured briquettes produced from different binders.

	% composition	Density (g/cm^3^)			
		Initial	30 min	60 min	120 min
Pitch	3	1.49	1.31	1.26	1.26
	5	1.48	1.34	1.25	1.24
	10	1.48	1.37	1.25	1.21
	15	1.49	1.36	1.25	1.19
	20	1.48	1.42	1.25	1.19
Molasses	3	1.51	1.22	1.28	1.32
	5	1.54	1.32	1.29	1.28
	10	1.55	1.32	1.33	1.29
	15	1.57	1.39	1.35	1.34
	20	1.58	1.43	1.36	1.38
Starch	3	1.46	1.29	1.26	1.24
	5	1.52	1.28	1.34	1.23
	10	1.55	1.28	1.31	1.27
	15	1.56	1.32	1.34	1.34
	20	1.57	1.33	1.37	1.35
Blended	B1	1.43	-	1.31	1.21
	B2	1.46	-	1.30	1.27
	B3	1.39	-	1.27	1.26
	B4	1.50	-	1.30	1.29

### 3.3 Cold crushing strength

**Fig. 1:**
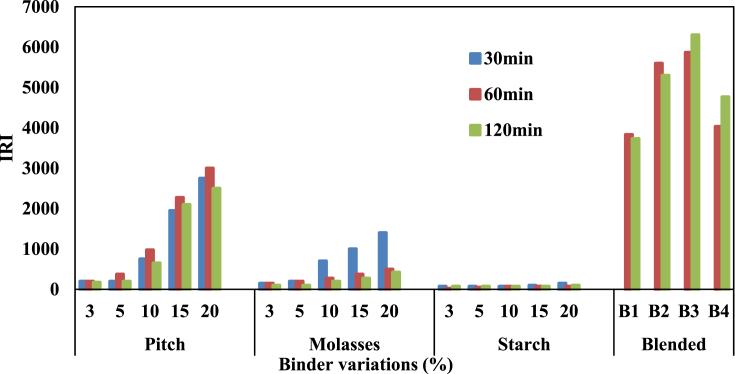
IRI of the briquettes produced from different binders and cured at 300 °C.

Table 3 represents the influence of varied percentage of binder contents on CCS of briquettes under different curing conditions (curing time, 300 °C, holding time of 30–120 min). Increase in binder content with 5% biochar addition led to an improved CCS for the briquettes. The CCS of pitch-bonded briquettes increased with an increase in holding time. This phenomenon is different for molasses-bonded briquettes. The CCS of the briquettes declined with increasing holding time. Starch binder gives the lowest CCS under different holding time. Molasses-bonded briquettes yielded the highest CCS even at 10% content. The interaction of biochar and molasses favored the CCS of the briquettes; however, the briquettes have low impact resistance (in terms of drop to fracture and IRI) compared to pitch-bonded briquettes as shown in Table 4 and Fig. 1. Briquette mechanical integrity cannot be limited to its CCS. It also depends on the ability to resist drop and impact force in conveyor belt during transfer, from chutes into bins, and off trucks onto the ground; thus, the idea of blending both binders along with biochar and coal slack. Briquettes produced from 5% pitch and 10% molasses gave the best CCS compared with others. All the briquettes produced in this study surpassed the benchmark of 350 kPa CCS suggested for briquettes of industrial application [17, 23]. The 350 kPa CCS benchmark has been found to be inadequate when it comes to operations with higher temperature burden where it can be employed as both fuel and reductant. Briquettes considered useful for metallurgical application must have CCS in the range of 6.9–30 MPa for blast furnace coke [2]. Briquettes with lower CCS will ultimately failed the purpose of its production due to the mechanical stresses and thermal pressure in whatever area of its application [25, 26]. Briquettes produced from 10 - 20% molasses, 8P-7M (B2) and 5P-10M (B4) have CCS higher than the lower end (6.9 MPa) of the mechanical strength of conventional coke. Compared to the highest CCS reported for briquettes produced from raw biomass and coal (847.50 kPa) [2], the CCS of the present samples are higher in all ranks including starch-bonded briquettes. The difference may be due to the initial dehydration and devolatilization carried out on biomass adopted for this study. It avoids the briquettes the tendencies of high devolatilization that can lead to crack formation within and at its surface. High hemicellulose and cellulose contents of biomass lead to rapid devolatilization even at low temperature curing thus leaving large pore volume and increase interstitial space between coal to coal and coal to biochar particles. However, biomass losses some portion of hemicellulose and cellulose during torrefaction process. This led to loss of dilute tar and light volatiles that can incur cracks during curing process leaving behind higher lignin tar in biochar that enhances bonding of coal slacks along with other binders [15].

**Table 3:** Cold crushing strength of briquettes produced using different binders and cured at 300°C.

	Pitch			Molasses			Starch			Blended		
Binder variation (%)	30 min	60 min	120 min	30 min	60 min	120 min	30 min	60 min	120 min		60 min	120 min
**3**	2.20	2.23	2.38	2.67	2.52	2.37	0.89	0.89	0.89	**B1**	3.25	5.18
**5**	2.31	2.45	2.69	4.72	4.05	3.59	1.34	1.22	1.11	**B2**	8.32	8.25
**10**	3.95	4.18	3.85	7.22	7.01	6.95	2.20	1.87	1.21	**B3**	5.39	5.47
**15**	4.24	4.66	4.64	7.76	7.39	7.02	2.43	1.89	1.68	**B4**	9.01	8.60
**20**	4.76	4.85	5.28	7.88	7.50	7.10	2.46	2.20	1.79

**Table 4:** Drop to fracture number of the briquettes produced from different binders.

	Pitch			Molasses			Starch			Blended		
Binder variation (%)	30 min	60 min	120 min	30 min	60 min	120 min	30 min	60 min	120 min		60 min	120 min
**3**	8	8	7	6	6	4	3	2	3	**B1**	115	112
**5**	8	15	8	8	8	4	3	2	3	**B2**	168	159
**10**	30	39	26	28	11	8	3	3	3	**B3**	176	189
**15**	78	91	84	40	15	11	4	3	3	**B4**	121	143
**20**	86	120	100	56	20	17	6	3	4

### 3.4 Drop to fracture and impact resistance

The impact resistance index (IRI) and drop resistance as observed in the pattern on Fig. 1 and Table 4, respectively, are intertwined into each other. Briquette that fails to meet the IRI target of 50 is not suitable for industrial and domestic applications [17]. The drop to fracture (times/2m) of pitch-bonded briquettes were higher than molasses-bonded and starch-bonded briquettes at every percentage composition. Richard [17] stated that direct relationship between some of the physical properties such as CCS, IRI and drop to fracture is expected. The claim is correct for briquettes made of the same binders. High CCS implied high IRI for briquettes produced with pitch, molasses and starch binder separately, however, the CCS and IRI of briquettes produced from the blended binder (pitch-molasses) cannot be directly compared.

Molasses-bonded briquettes are higher in CCS compared with pitch-bonded; however, the drop to fracture and IRI of pitch bonded briquettes are much higher than that of molasses. The drop to fracture of pitch-bonded briquettes was higher at 60 min holding time as against 30 min of molasses. The drops to fracture of pitch-molasses bonded briquettes were greater than 100 (time/2m). The drop to facture obtained for 15–20% molasses, pitch and pitch-molasses bonded briquettes were better than 56.6 (times/2m) reported for briquettes produced from high volatile coal using 13%pitch-2%molasses for COREX iron making process [1]. It is an indication that lean grade coal slacks briquettes from the present study can substantially be useful as feedstock for metallurgical applications such as COREX iron making and rotary kiln DRI.

Except for starch-bonded where IRI were as low as 25 for 3–5% starch composition, the other briquettes crossed the IRI target recommended for industrial briquettes. Carbon briquettes prepared from low ranked coal was reported to have the highest IRI value of 650 by Lázaro et al. [27]. Another effort by Blesa et al. [8] recorded a IRI up to 1000 for smokeless briquettes produced from pyrolyzed (500–700 °C) low ranked coal and sawdust/or olive stone. Based on the IRI, the briquettes produced in the present study are better in mechanical integrity. The pitch-molasses bonded briquettes were the result of the observation of the pitch and molasses bonded briquettes. Both CCS and IRI are important physical properties of the briquettes and pitch-molasses bonded briquettes possess both at an elevated value far more than required. It shows that the pitch-molasses blend along with biochar can improve the physical properties of coal slack briquettes.

### 3.5 Water resistance

**Fig. 2:**
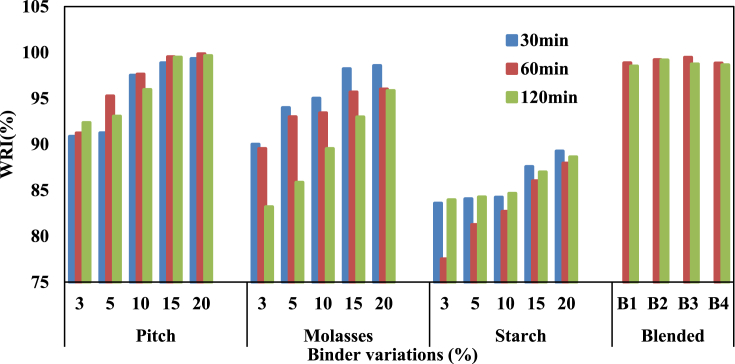
WRI of the briquettes produced from various binders and cured at 300 °C.

Binder-less coal briquettes made from high pressure have been reported to usually be resistant to water absorption. However due to utilization of binders that can be water-sensitive, water resistance index (WRI) was benched at 95% [17]. Pitch-bonded briquettes (10–15%) have WRI greater than 95% benchmark as shown in Fig. 2. Briquettes from 3 - 10% molasses binder failed to reach this benchmark, however at 15–20% addition, the briquettes surpassed 95%. Starch-bonded briquettes generally failed to meet this requirement under all curing conditions (75–90%). The blends of pitch-molasses bonded briquettes surpassed the benchmark as shown on Fig. 2. The WRI results obtained in this study are higher than the highest value of 90% reported by Zarringhalam-Moghaddam et al. [2] for coal-biomass briquettes. It was observed that the bonding strength of the briquettes is responsible for its water absorption or repelling property. Good bonding creates lesser pores as against larger pores for weaker briquettes, thus limiting the quantity of water that was absorbed. Curing conditions also played a significant role since higher holding time leads to higher pore/crack formation within the briquettes. Briquettes produced from the blended binder along with biochar are thus far preferred to briquettes from each binder due to its excellent properties.

### 3.6 Proximate, ultimate and calorific value analyses of briquettes

Table 5 represents the influence of binder types on the proximate, ultimate and gross calorific values of the composite fuel (briquettes). The combustion properties of the briquettes produced from pitch, molasses, starch and the blend of pitch and molasses were similar to that of raw coal slacks with some minor increment in the carbon content and about 8% addition to the calorific values. The variations in binder content do not cause so much disparity to the proximate contents, ultimate contents and gross calorific values of the composite fuel. The behavior of the binders (organic) confirms previous assertion that they have infinitesimal impact on the combustion properties of briquettes when used for bonding of coal [1, 4]. Therefore, the organic binders do not cause deterioration to the combustion properties of the composite fuel.

**Table 5:** Proximate, ultimate and calorific value of briquette samples.

	BV (%)	Proximate analysis (%)				Ultimate analysis (%)					GCV
		MC	VM	AC	FC	C	H	N	S	O	(MJ/kg)
Pitch	**3**	1.51	14.18	18.03	66.28	75.56	2.18	0.89	0.71	20.66	25.22
	**5**	1.52	14.18	18.03	66.27	75.57	2.20	0.90	0.70	20.63	25.23
	**10**	1.51	14.20	18.03	66.26	75.60	2.21	0.92	0.72	20.55	25.19
	**15**	1.53	14.21	18.03	66.23	75.60	2.20	0.89	0.71	20.60	25.20
	**20**	1.55	14.21	18.03	66.21	75.61	2.19	0.88	0.72	20.60	25.24
Molasses	**3**	1.62	13.65	18.08	66.65	75.13	2.20	0.97	0.72	20.98	25.01
	**5**	1.63	13.66	18.08	66.63	75.13	2.18	0.96	0.71	21.02	25.00
	**10**	1.63	13.63	18.08	66.66	75.14	2.18	0.98	0.72	20.98	24.96
	**15**	1.64	13.42	18.08	66.86	75.15	2.19	0.97	0.71	20.98	24.92
	**20**	1.64	13.28	18.08	67.00	75.16	2.20	0.98	0.72	20.95	24.88
Starch	**3**	1.60	13.85	18.04	66.51	75.15	2.10	0.93	0.71	21.11	25.02
	**5**	1.60	13.85	18.04	66.51	75.16	2.12	0.94	0.72	21.06	25.01
	**10**	1.61	13.86	18.04	66.49	75.16	2.12	0.94	0.72	21.06	25.02
	**15**	1.61	13.86	18.04	66.49	75.17	2.13	0.93	0.73	21.04	25.00
	**20**	1.62	13.88	18.04	66.49	75.18	2.10	0.94	0.73	21.05	25.06
Blended	**B1**	1.61	13.38	18.05	66.96	75.20	2.13	0.88	0.72	21.07	25.04
	**B2**	1.58	13.48	18.05	66.89	75.25	2.15	0.89	0.73	20.99	25.14
	**B3**	1.63	13.36	18.03	66.98	75.36	2.15	0.91	0.74	20.84	25.22
	**B4**	1.61	13.30	18.04	67.03	75.34	2.16	0.92	0.73	20.85	24.96

*BV-Binder variation.

### 3.7 Microstructure and elemental composition of the briquettes

**Fig. 3:**
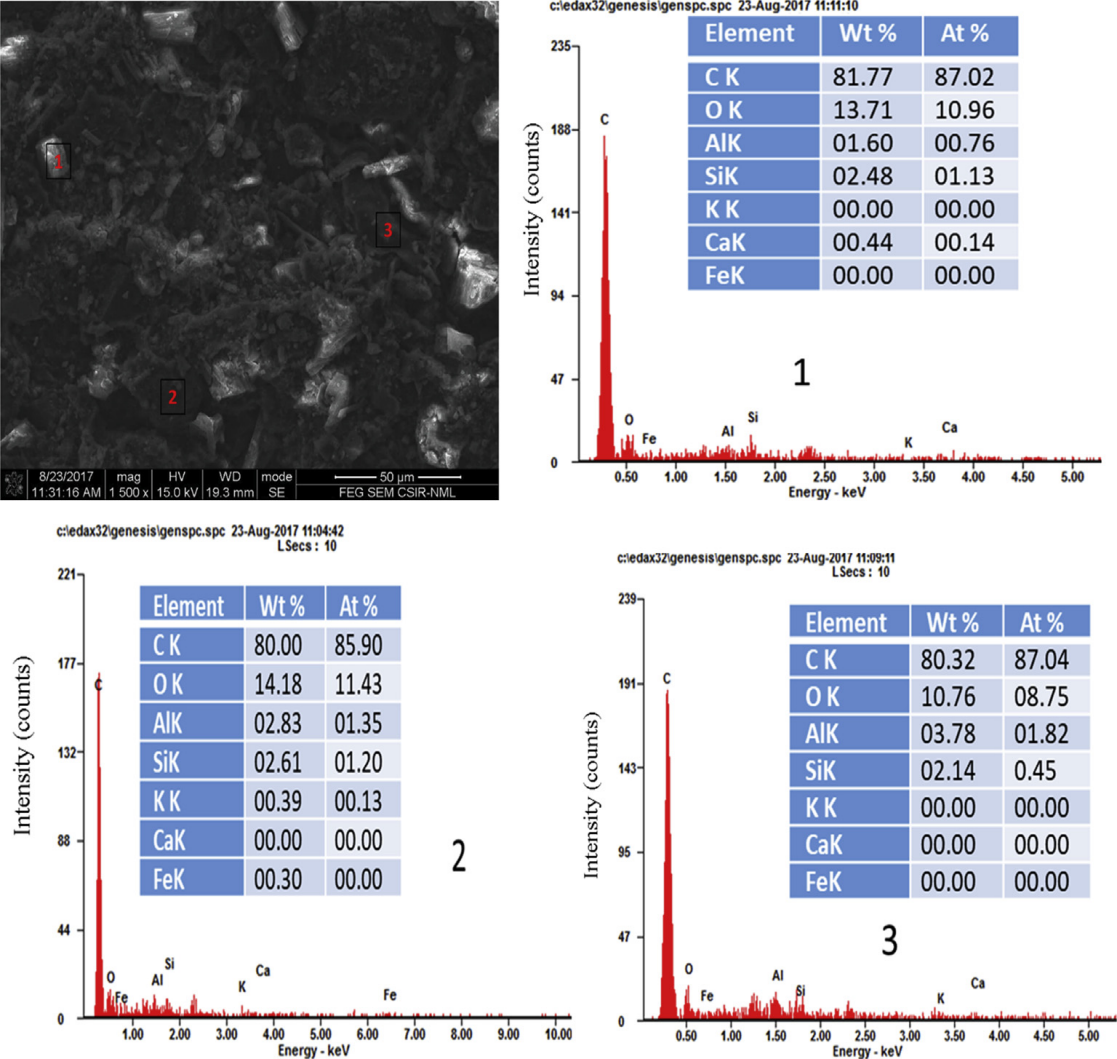
Micrograph and the EDX spectra of points (1–3) for B2 (120 min).

**Fig. 4:**
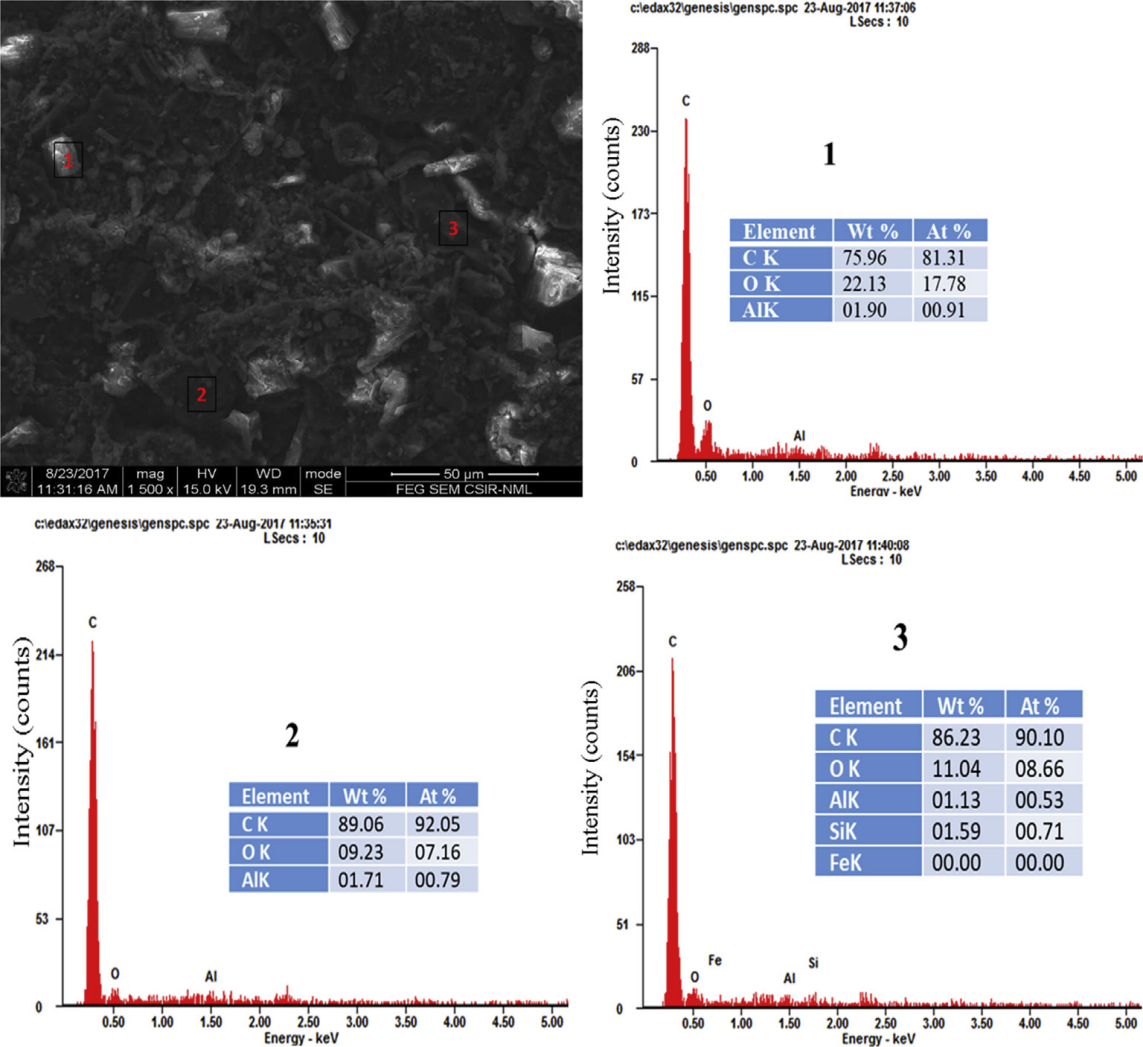
Micrograph and the EDX spectra of points (1–3) for B4 (120 min).

Figs. 3 and 4 represent microstructures and elemental composition of the briquettes produced from two of the blends of pitch and molasses. Grainy irregular surface associated with some fissures can be observed on the micrographs. The moisture and water of molasses were evaporated after the curing process. Therefore, the dry pitch, molasses along with sufficient amount of oxygen bridges were left to bond the coal and biochar particles into a strong briquette. The energy spectrum analysis results in Figs. 3 and 4 (points 1, 2, and 3) are similar except that oxygen content in Fig. 4 (point 1). The elemental composition of the briquettes remains unchanged with the addition of the blended binder. The briquettes consist primarily consist of C, oxygen, Al, Ca and Si.

## 4 Conclusion

The densification of coal fines and biochar using different organic binders and their blends has been carried out. Briquettes produced from single binder satisfied CCS, drop to fracture, IRI, and WRI at 15–20% satisfactorily. Moreover, briquettes produced from coal fines, biochar and blend of pitch-molasses (15%) were better with IRI of 6300, CCS of 9 MPa, drop to fracture greater than 100 (times/2m) and 99%WRI. The proximate, ultimate and calorific values of the briquettes did not deteriorate compared with that coal fines. The coal fines-biochar briquettes satisfactorily met required physical properties for metallurgical coke and thus can not only be adopted as source of fuel and also as reductant in metallurgical operations such as Rotary kiln DRI production scheme and COREX iron-making process.

## Declarations

### Author contribution statement

Adekunle Adeleke: Conceived and designed the experiments; Performed the experiments; Analyzed and interpreted the data; Contributed reagents, materials, analysis tools or data; Wrote the paper.

Jamiu Odusote, O. Lasode: Conceived and designed the experiments.

Peter Ikubanni: Analyzed and interpreted the data; Contributed reagents, materials, analysis tools or data; Wrote the paper.

Malathi Madhurai, D. Paswan: Performed the experiments.

### Funding statement

This work was supported by The World Academy of Science (TWAS), Italy and Council for Scientific and Industrial Research (CSIR), India.

### Competing interest statement

The authors declare no conflict of interest.

### Additional information

No additional information is available for this paper.

## References

[1] Zhong Q., Yang Y., Li Q., Xu B., Jiang T. (2017). Coal tar pitch and molasses blended binder for production of formed coal briquettes from high volatile coal. Fuel Process. Technol..

[2] Zarringhalam-Moghaddam Gholipour-Zanjani N., Dorosti S., Vaez M. (2011). Physical properties of solid fuel briquettes from bituminous coal waste and biomass. J. Coal Sci. Eng..

[3] Massaro M.M., Son S.F., Groven L.J. (2014). Mechanical, pyrolysis, and combustion charac- terization of briquetted coal fines with municipal solid waste plastic (MSW) binders. Fuel.

[4] Mollah M.M., Jackson W.R., Marshall M., Chaffee A.L. (2015). An attempt to produce blast furnace coke from Victorian brown coal. Fuel.

[5] Taulbee D., Patil D.P., Honaker R.Q., Parekh B.K. (2009). Briquetting of coal fines and sawdust Part I: binder and briquetting-parameters evaluation. Int. J.Coal Prep.Util..

[6] Benk A., Coban A. (2011). Investigation of resole, novalac and coal tar pitch blended binder for the production of metallurgical quality formed coke briquettes from coke breeze and anthracite. Fuel Process. Technol..

[7] Steel Tata (2017). Briquetting of Coal Fines. http://www.tatainnoverse.com/challenge.php?id=39.

[8] Blesa M.J., Miranda J.L., Izquierdo M.T., Moliner R. (2003). Curing temperature effect on mechanical strength of smokeless fuel briquettes prepared with molasses. Fuel.

[9] Adeleke A.A., Odusote J.K., Lasode O.A., Ikubanni P.P., Malathi M., Paswan D. (2019). Mild pyrolytic treatment of gmelina arborea for optimum energetic yields. Cogent Eng..

[10] Odusote J.K., Adeleke A.A., Lasode O.A., Malathi M., Paswan D. (2019). Thermal and Compositional Properties of Treated Tectona Grandis.

[11] (1984). IS: 1350-1. Indian Standard Methods of Test for Coal and Coke, Part 1: Proximate Analysis PCD 7: Solid mineral Fuels, Reaffirmed in 2002.

[12] ASTM D5373-16 (2016). Standard Test Methods for Determination of Carbon, Hydrogen and Nitrogen in Analysis Samples of Coal and Carbon in Analysis Samples of Coal and Coke.

[13] ASTM D5865-04 (2004). Standard Test Method for Gross Calorific Value of Coal and Coke.

[14] Zhong Q., Yang Y., Jiang T., Li Q., Xu B. (2016). Xylene activation of coal tar pitch binding characteristics for production of metallurgical quality briquettes from coke breeze. Fuel Process. Technol..

[15] Lumadue M.R., Cannon F.S., Brown N.R. (2012). Lignin as both fuel and fusing binder in briquetted anthracite fines for foundry coke substitute. Fuel.

[16] Wang P.F., Jin L.J., Liu J.H., Zhu S.W., Hu H.Q. (2013). Analysis of coal tar derived frompyrol- ysis at different atmospheres. Fuel.

[17] Richard S.R. (1990). Briquetting peat and peat - coal mixtures. Fuel Process. Technol..

[18] Tembe E.T., Otache P., Ekhuemelo D. (2014). Density, Shatter index, and combustion properties of briquettes produced from groundnut shells, rice husks and sawdust of Daniella Oliveri. J.Appl.Biosci..

[19] Balogun A.O., Lasode O.A., McDonald A.G. (2014). Thermo-analytical and physico-chemical characterization of woody and non-woody biomass from an agro-ecological zone in Nigeria. BioResources.

[20] Lasode O.A., Balogun A.O., McDonald A.G. (2014). Torrefaction of some Nigerian lignocellulosic resources, 2014 resources and decomposition kinetics. J. Anal. Appl. Pyrolysis.

[21] Dhungana A., Dutta A., Basu P. (2012). Torrefaction of non-lignocellulose biomass waste. Can. J. Chem. Eng..

[22] van der Stelt M.J.C., Gerhauser H., Kiel J.H.A., Ptasinski K.J. (2011). Biomass upgrading by torrefaction for the production of biofuels: a review. Biomass Bioenergy.

[23] Thoms L.J., Snape C.E., Taylor D. (1999). Physical characteristics of cold cured anthracite/coke breeze briquettes prepared from a coal tar acid resin. Fuel.

[24] Mori A., Kubo S., Kudo S., Norinaga K., Kanai T., Aoki H. (2011). Preparation of high-strength coke by carbonization of hot-briquetted Victorian brown coal. Energy Fuels.

[25] Paul S.A., Hull A.S., Plancher H., Agarwal P.K. (2002). Use of asphalts for formcoke briquettes. Fuel Process. Technol..

[26] Yip K., Wu H., Zhang D.K. (2007). Pyrolysis of Collie coal briquettes to produce char as a metallurgical reductant. Energy Fuels.

[27] Lázaro M.J., Boyano A., Gálvez M.E., Izquierdo M.T., Moliner R. (2007). Low-cost carbon-based briquettes for the reduction of no emissions from medium-small stationary sources. Catal. Today.

